# Correction: X-ray wavefunction refinement and comprehensive structural studies on bromo-substituted analogues of 2-deoxy-d-glucose in solid state and solution

**DOI:** 10.1039/d2ra90029g

**Published:** 2022-04-01

**Authors:** Marcin Ziemniak, Sylwia Pawlędzio, Anna Zawadzka-Kazimierczuk, Paulina M. Dominiak, Damian Trzybiński, Wiktor Koźmiński, Rafał Zieliński, Izabela Fokt, Waldemar Priebe, Krzysztof Woźniak, Beata Pająk

**Affiliations:** Biological and Chemical Research Centre, Department of Chemistry, University of Warsaw Zwirki i Wigury 101 02-089 Warszawa Poland mziemniak@chem.uw.edu.pl spawledzio@chem.uw.edu.pl anzaw@chem.uw.edu.pl dtrzybinski@chem.uw.edu.pl kozmin@chem.uw.edu.pl kwozniak@chem.uw.edu.pl; Department of Experimental Therapeutics, The University of Texas MD Anderson Cancer Center 1901 East Rd. Houston TX 77054 USA rziel77@gmail.com ifokt@mdanderson.org wpriebe@mac.com; Independent Laboratory of Genetics and Molecular Biology, Kaczkowski Military Institute of Hygiene and Epidemiology Kozielska 4 01-163 Warsaw Poland bepaj@wp.pl

## Abstract

Correction for ‘X-ray wavefunction refinement and comprehensive structural studies on bromo-substituted analogues of 2-deoxy-d-glucose in solid state and solution’ by Marcin Ziemniak *et al.*, *RSC Adv.*, 2022, **12**, 8345–8360. DOI: 10.1039/D1RA08312K

The authors regret that the name of one of the authors (Anna Zawadzka-Kazimierczuk) was shown incorrectly in the original article. The corrected author list is as shown above.

The Royal Society of Chemistry apologises for these errors and any consequent inconvenience to authors and readers.

## Supplementary Material

